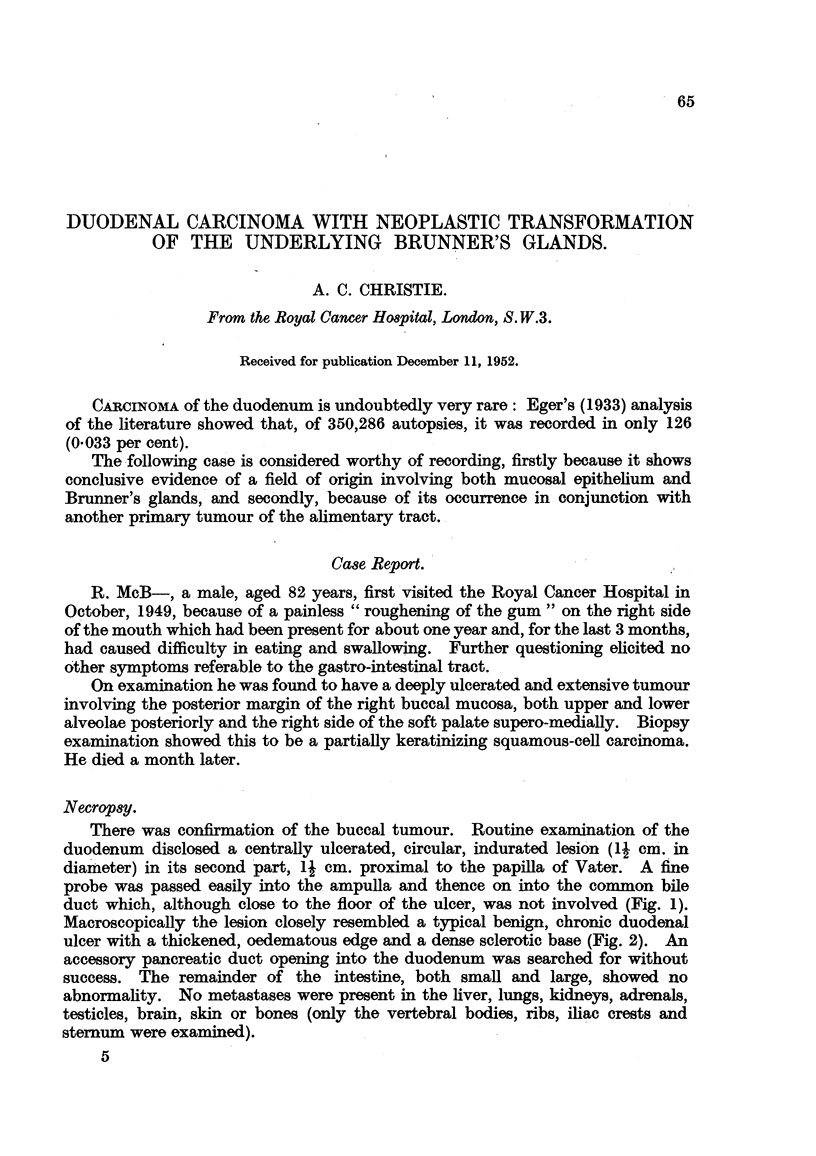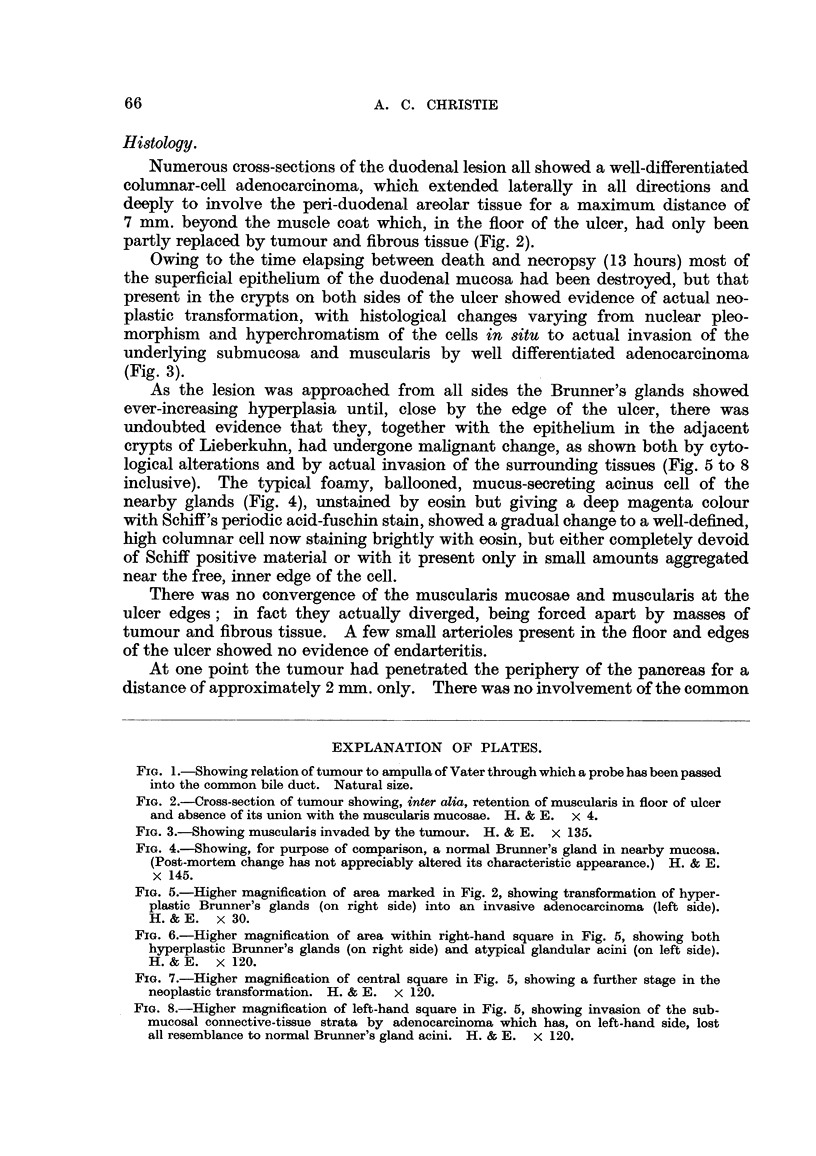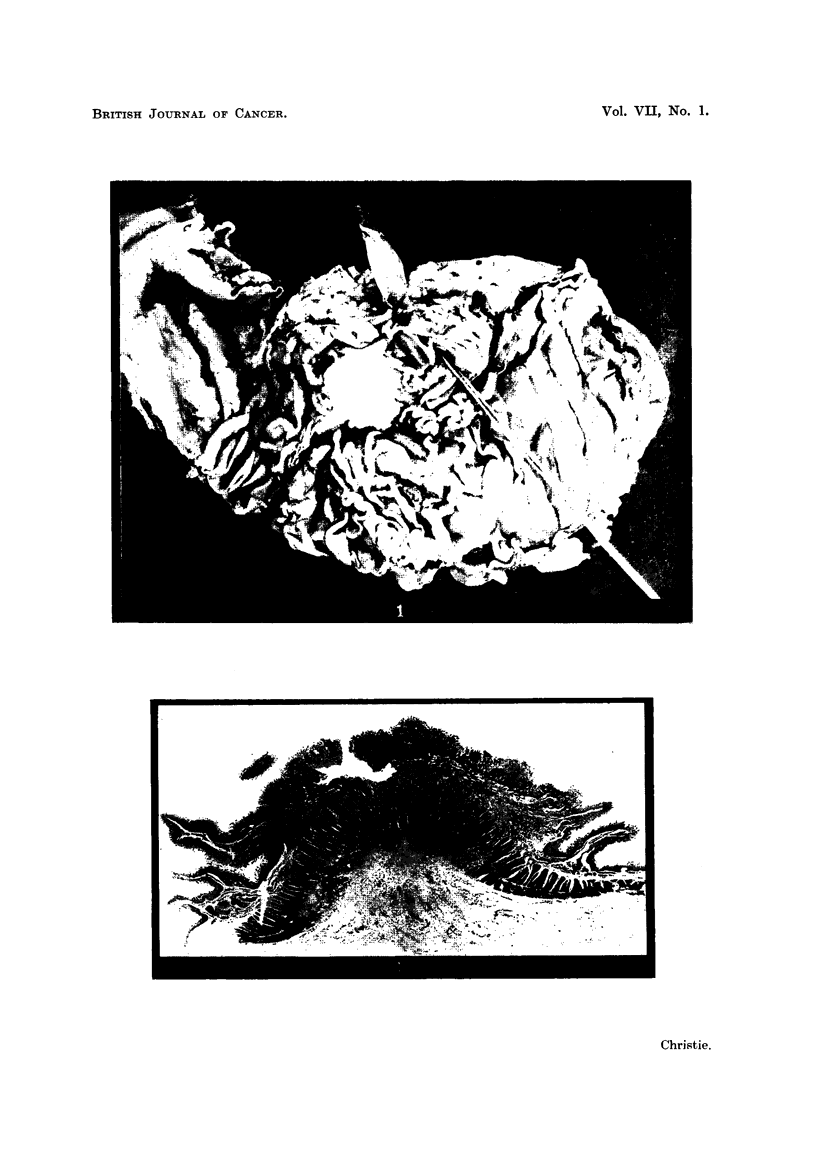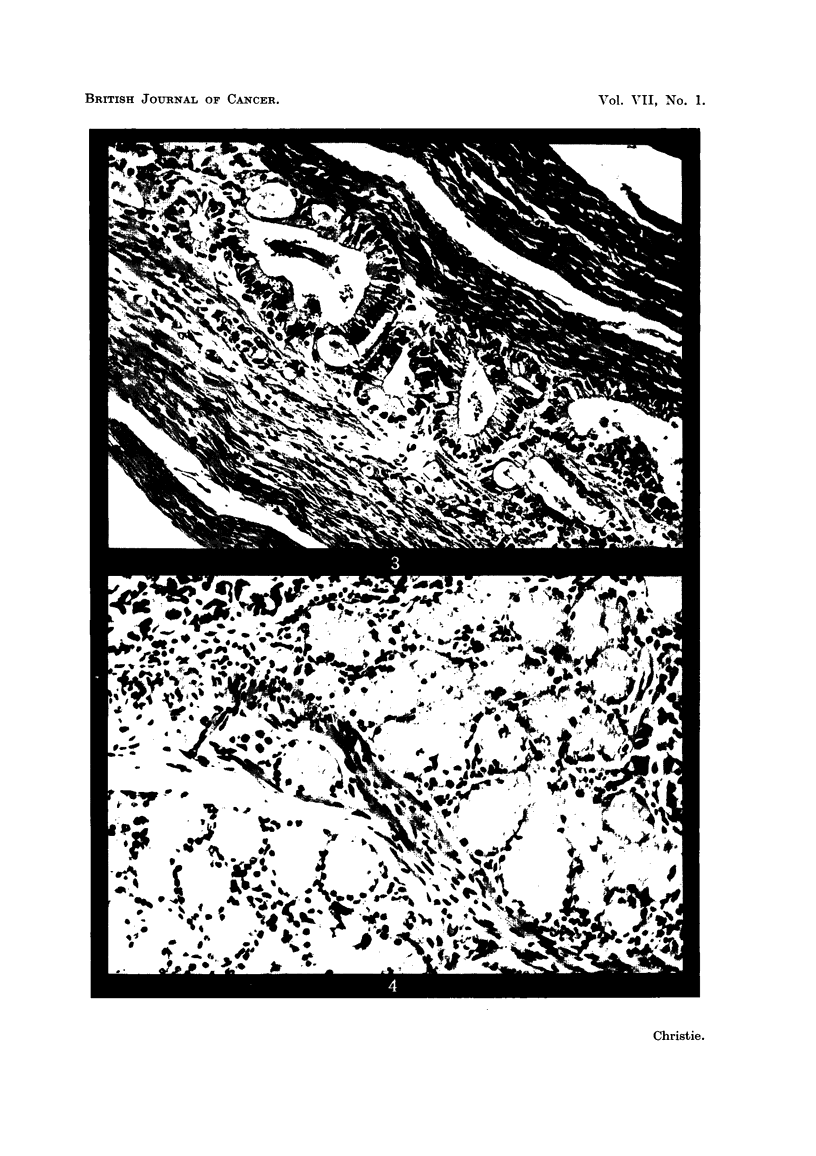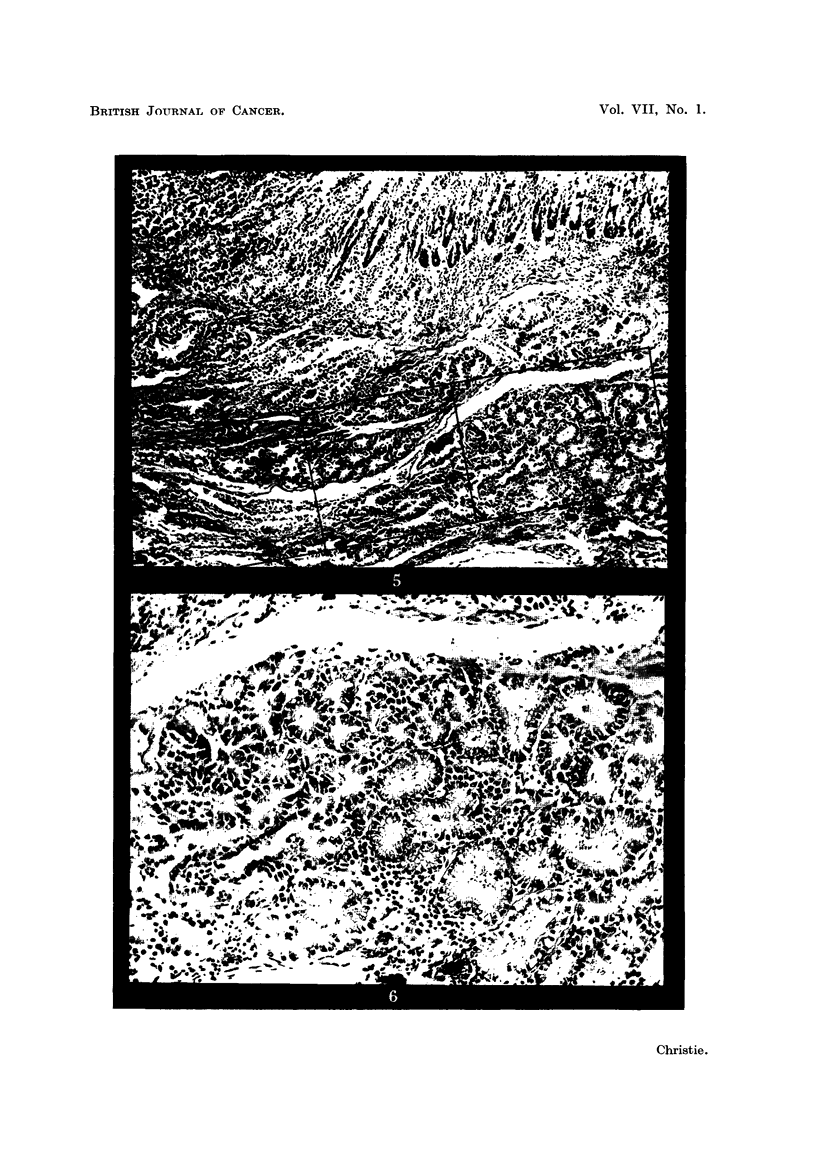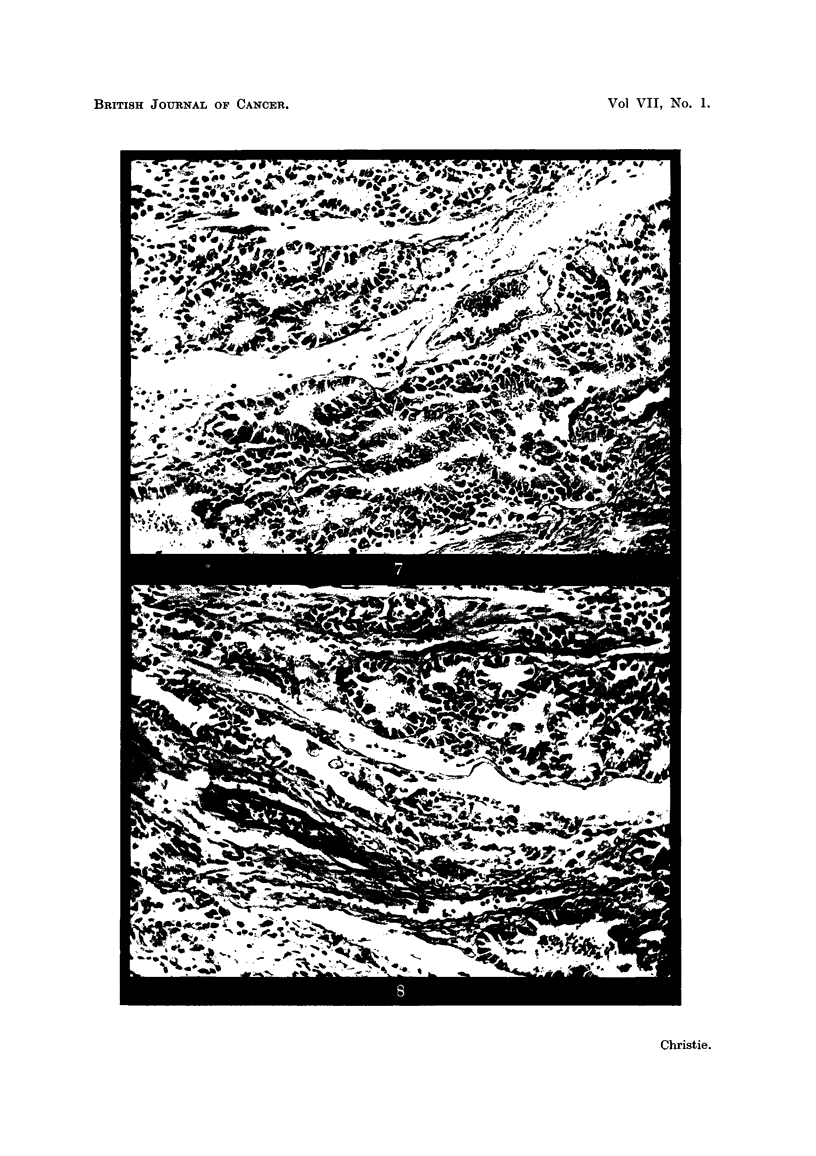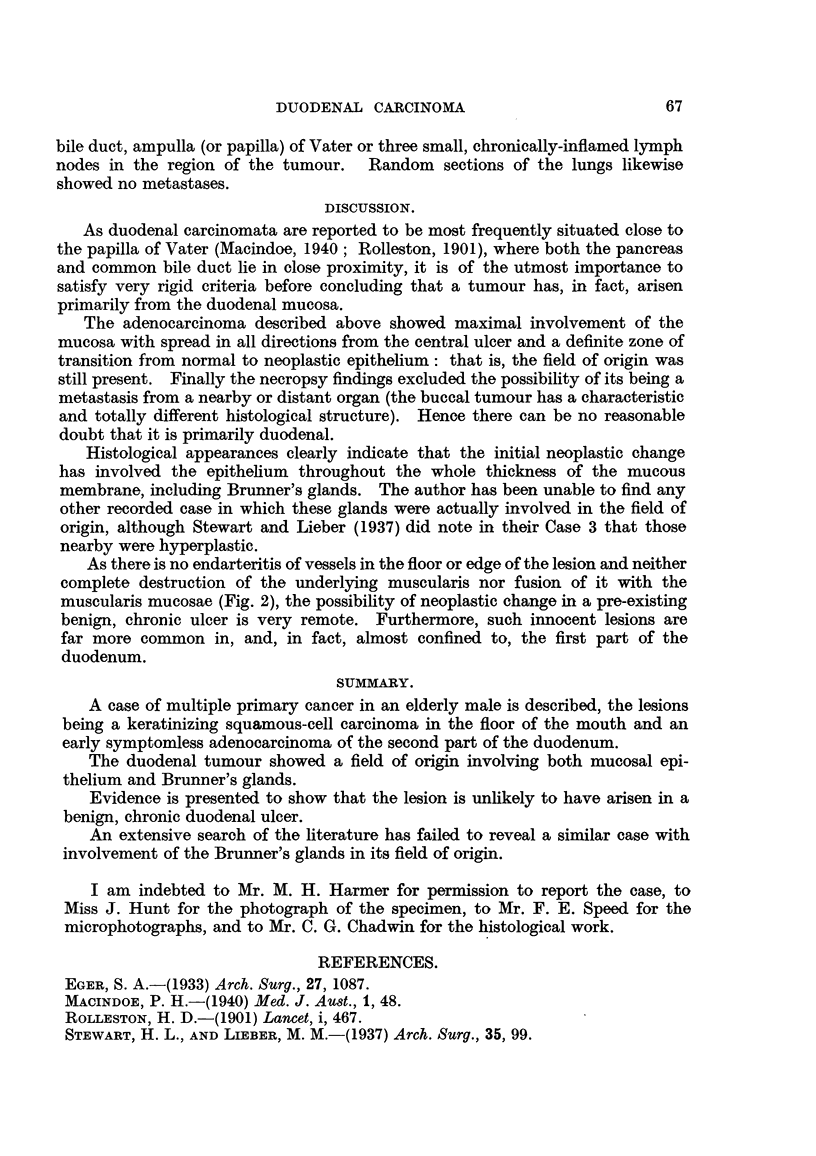# Duodenal Carcinoma with Neoplastic Transformation of the Underlying Brunner's Glands

**DOI:** 10.1038/bjc.1953.7

**Published:** 1953-03

**Authors:** A. C. Christie

## Abstract

**Images:**


### DUODENAL CARCINOMA WITH NEOPLASTIC TRANSFORMATION OF THE UNDERLYING BRUNNER'S GLANDS.

A. C. CHRISTIE.

*From the Royal Cancer Hospital, London, S.W.3.*

Received for publication December 11, 1952.

CARCINOMA of the duodenum is undoubtedly very rare : Eger's (1933) analysis of the literature showed that, of 350,286 autopsies, it was recorded in only 126 (0·033 per cent).

The following case is considered worthy of recording, firstly because it shows conclusive evidence of a field of origin involving both mucosal epithelium and Brunner's glands, and secondly, because of its occurrence in conjunction with another primary tumour of the alimentary tract.

### *Case Report.*

R. McB—, a male, aged 82 years, first visited the Royal Cancer Hospital in October, 1949, because of a painless " roughening of the gum " on the right side of the mouth which had been present for about one year and, for the last 3 months, had caused difficulty in eating and swallowing. Further questioning elicited no other symptoms referable to the gastro-intestinal tract.

On examination he was found to have a deeply ulcerated and extensive tumour involving the posterior margin of the right buccal mucosa, both upper and lower alveolae posteriorly and the right side of the soft palate supero-medially. Biopsy examination showed this to be a partially keratinizing squamous-cell carcinoma. He died a month later.

### *Necropsy.*

There was confirmation of the buccal tumour. Routine examination of the duodenum disclosed a centrally ulcerated, circular, indurated lesion (1½ cm. in diameter) in its second part, 1½ cm. proximal to the papilla of Vater. A fine probe was passed easily into the ampulla and thence on into the common bile duct which, although close to the floor of the ulcer, was not involved (Fig. 1). Macroscopically the lesion closely resembled a typical benign, chronic duodenal ulcer with a thickened, oedematous edge and a dense sclerotic base (Fig. 2). An accessory pancreatic duct opening into the duodenum was searched for without success. The remainder of the intestine, both small and large, showed no abnormality. No metastases were present in the liver, lungs, kidneys, adrenals, testicles, brain, skin or bones (only the vertebral bodies, ribs, iliac crests and sternum were examined).

*Histology.*

Numerous cross-sections of the duodenal lesion all showed a well-differentiated columnar-cell adenocarcinoma, which extended laterally in all directions and deeply to involve the peri-duodenal areolar tissue for a maximum distance of 7 mm. beyond the muscle coat which, in the floor of the ulcer, had only been partly replaced by tumour and fibrous tissue (Fig. 2).

Owing to the time elapsing between death and necropsy (13 hours) most of the superficial epithelium of the duodenal mucosa had been destroyed, but that present in the crypts on both sides of the ulcer showed evidence of actual neoplastic transformation, with histological changes varying from nuclear pleomorphism and hyperchromatism of the cells *in situ* to actual invasion of the underlying submucosa and muscularis by well differentiated adenocarcinoma (Fig. 3).

As the lesion was approached from all sides the Brunner's glands showed ever-increasing hyperplasia until, close by the edge of the ulcer, there was undoubted evidence that they, together with the epithelium in the adjacent crypts of Lieberkuhn, had undergone malignant change, as shown both by cytological alterations and by actual invasion of the surrounding tissues (Fig. 5 to 8 inclusive). The typical foamy, ballooned, mucus-secreting acinus cell of the nearby glands (Fig. 4), unstained by eosin but giving a deep magenta colour with Schiff's periodic acid-fuschin stain, showed a gradual change to a well-defined, high columnar cell now staining brightly with eosin, but either completely devoid of Schiff positive material or with it present only in small amounts aggregated near the free, inner edge of the cell.

There was no convergence of the muscularis mucosae and muscularis at the ulcer edges; in fact they actually diverged, being forced apart by masses of tumour and fibrous tissue. A few small arterioles present in the floor and edges of the ulcer showed no evidence of endarteritis.

At one point the tumour had penetrated the periphery of the pancreas for a distance of approximately 2 mm. only. There was no involvement of the common

---

EXPLANATION OF PLATES.

Fig. 1.—Showing relation of tumour to ampulla of Vater through which a probe has been passed into the common bile duct. Natural size.

Fig. 2.—Cross-section of tumour showing, *inter alia*, retention of muscularis in floor of ulcer and absence of its union with the muscularis mucosae. H. & E. × 4.

Fig. 3.—Showing muscularis invaded by the tumour. H. & E. × 135.

Fig. 4.—Showing, for purpose of comparison, a normal Brunner's gland in nearby mucosa. (Post-mortem change has not appreciably altered its characteristic appearance.) H. & E. × 145.

Fig. 5.—Higher magnification of area marked in Fig. 2, showing transformation of hyperplastic Brunner's glands (on right side) into an invasive adenocarcinoma (left side). H. & E. × 30.

Fig. 6.—Higher magnification of area within right-hand square in Fig. 5, showing both hyperplastic Brunner's glands (on right side) and atypical glandular acini (on left side). H. & E. × 120.

Fig. 7.—Higher magnification of central square in Fig. 5, showing a further stage in the neoplastic transformation. H. & E. × 120.

Fig. 8.—Higher magnification of left-hand square in Fig. 5, showing invasion of the submucosal connective-tissue strata by adenocarcinoma which has, on left-hand side, lost all resemblance to normal Brunner's gland acini. H. & E. × 120.

1

2

3

4

Christie.

5

6

Christie.

7

8

Christie.

bile duct, ampulla (or papilla) of Vater or three small, chronically-inflamed lymph nodes in the region of the tumour. Random sections of the lungs likewise showed no metastases.

### DISCUSSION.

As duodenal carcinomata are reported to be most frequently situated close to the papilla of Vater (Macindoe, 1940 ; Rolleston, 1901), where both the pancreas and common bile duct lie in close proximity, it is of the utmost importance to satisfy very rigid criteria before concluding that a tumour has, in fact, arisen primarily from the duodenal mucosa.

The adenocarcinoma described above showed maximal involvement of the mucosa with spread in all directions from the central ulcer and a definite zone of transition from normal to neoplastic epithelium : that is, the field of origin was still present. Finally the necropsy findings excluded the possibility of its being a metastasis from a nearby or distant organ (the buccal tumour has a characteristic and totally different histological structure). Hence there can be no reasonable doubt that it is primarily duodenal.

Histological appearances clearly indicate that the initial neoplastic change has involved the epithelium throughout the whole thickness of the mucous membrane, including Brunner's glands. The author has been unable to find any other recorded case in which these glands were actually involved in the field of origin, although Stewart and Lieber (1937) did note in their Case 3 that those nearby were hyperplastic.

As there is no endarteritis of vessels in the floor or edge of the lesion and neither complete destruction of the underlying muscularis nor fusion of it with the muscularis mucosae (Fig. 2), the possibility of neoplastic change in a pre-existing benign, chronic ulcer is very remote. Furthermore, such innocent lesions are far more common in, and, in fact, almost confined to, the first part of the duodenum.

### SUMMARY.

A case of multiple primary cancer in an elderly male is described, the lesions being a keratinizing squamous-cell carcinoma in the floor of the mouth and an early symptomless adenocarcinoma of the second part of the duodenum.

The duodenal tumour showed a field of origin involving both mucosal epithelium and Brunner's glands.

Evidence is presented to show that the lesion is unlikely to have arisen in a benign, chronic duodenal ulcer.

An extensive search of the literature has failed to reveal a similar case with involvement of the Brunner's glands in its field of origin.

I am indebted to Mr. M. H. Harmer for permission to report the case, to Miss J. Hunt for the photograph of the specimen, to Mr. F. E. Speed for the microphotographs, and to Mr. C. G. Chadwin for the histological work.

### REFERENCES.

Eger, S. A.—(1933) *Arch. Surg.*, **27**, 1087.
Macindoe, P. H.—(1940) *Med. J. Aust.*, **1**, 48.
Rolleston, H. D.—(1901) *Lancet*, i, 467.
Stewart, H. L., and Lieber, M. M.—(1937) *Arch. Surg.*, **35**, 99.